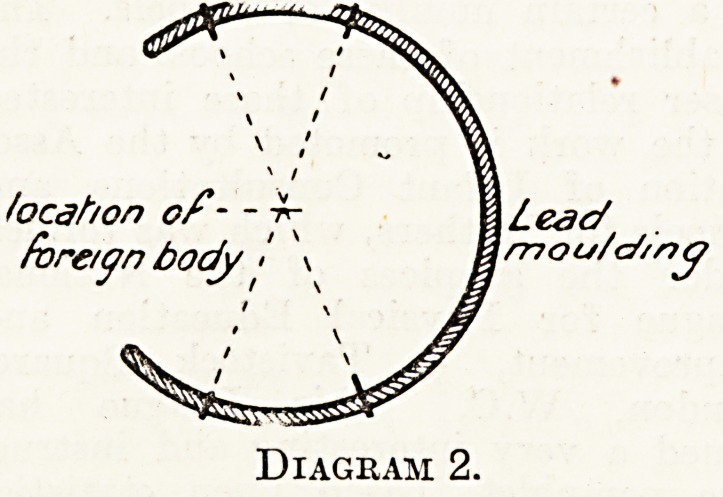# The Institutional Worker

**Published:** 1913-06-28

**Authors:** 


THE
### INSTITUTIONAL WORKER
**Being a Special Supplement to "The Hospital"**

### OUR BUREAU OF INFORMATION.

### Rules for Correspondents.

1. Every letter must be accompanied by the coupon to be cut from the back cover (inside page) of The Hospital, current issue, and must contain the name and address of the correspondent with pseudonym for publication if desired. Replies by post cannot be given, save under exceptional circumstances at the Editor's discretion.

2. Letters from Approved Homes in reply to special needs published in the Bureau should state terms and full particulars, and be sent prepaid under cover to the Editor of the Bureau with name written across coupon for identification.

3. Proprietors of Homes which have not yet been entered on the List of Approved Homes, but have spare accommodation likely to suit special needs, are invited to write for an application form for registration. The fee for registration, which includes two announcements of the Home in the Bureau and other privileges, is 10s.

4. All communications to be addressed to the Editor of The Hospital, 28 Southampton Street, Strand, London, W.C., and marked "Bureau of Information."

### *INSTITUTIONAL FACTS AND FIGURES.*

### Location of Foreign Bodies by X-Rays.

The following description of a simple and ingenious method of locating foreign bodies by *x*-rays, which originated at the Middlesex Hospital, will commend itself to *x*-ray operators, especially as it obviates the use of expensive apparatus :—

For purposes of description we will assume that it is desired to locate a foreign body in the patient's arm. Arrange the arm in a convenient position upon the *x*-ray couch, and place the tube beneath it. Screen in the ordinary way, and having ascertained the presence of the foreign body, insert a metal pointer beneath the screen and place the point on the skin surface of the limb proximal to the screen so that its shadow and that of the foreign body, projected on the screen, coincide. Remove the screen, and by the aid of a light, mark the spot indicated on the skin. Replace the screen, and in a similar manner mark the skin on the surface proximal to the couch at the point of coincidence of the shadows of the pointer and the foreign body. It is important that these two observations be taken without disturbing the position of the limb. The limb should now be rotated slightly, and two similar observations of the foreign body made in this position and marked

Diagram 1.

upon the skin. Thus four points will have been marked upon the skin—two upon the upper and two upon the lower surface of the limb, as indicated in Diagram 1. Next take a strip of lead, mould it carefully round the limb, and mark upon it the positions of the four points noted on the skin (see Diagram 1). Remove the mould carefully, and make a tracing of it on paper, marking, of course, the positions of the points. Join the upper and lower points by straight lines so that they intersect one another, as shown in Diagram 2; the point of intersection will indicate the position of the foreign

Diagram 2.

body, and the measurements from this point to the points at the circumference will indicate the depth of it from the corresponding markings on the skin surface.

### A Useful Laundry Machine.

The Troy Dryroom Tumbler, which is now largely in use in America, will be found a useful addition to the institutional laundry. The machine is an American invention of comparatively recent date, and was primarily designed for drying bath-towels, mats, and similar articles, which can best be done by the application of a blast of hot air to the goods whilst revolving in a woven wire cylinder, the principle embodied in the design. A more recent and very useful application of the machine is the drying of feather pillows. The pillows are washed in the regular way in the ordinary washer, and after the removal of excess of water in the hydro-extractor they are placed in the tumbler and dried. This renews the life of the feathers, and allows of their thorough cleansing without removal from the pillow-case. The machine is manufactured by the Troy Laundry Machinery Co., of Chicago and London.

### Sterilising Instruments.

For sterilising boilable instruments, sodium hydrate (caustic soda) in solution will be found preferable to carbonate of soda. Whereas instruments boiled in a solution of carbonate of soda, and more especially plated instruments, are liable to discolouration unless quickly dried, sodium hydrate, when used in 0.25 per cent. solution (about 38 grains to the quart of water), has not this disadvantage. Fresh blood-stains are quickly removed when rinsed in this solution, and older stains merely require a little rubbing in addition to rinsing. It is a good plan to rinse the instruments in the solution immediately after use, for much time will thus be saved, and scouring, which is so often resorted to, and which is so damaging to plated instruments, will not be necessary.

---

### *THE SICK AND DISTRESSED.*

**The Editor is prepared to make known without charge the needs of any who may be sick or in difficulties, and to guide them in making choice of Homes for treatment or convalescence. Reduced terms can often be arranged with Homes on the Approved List for those unable to pay full fees. Queries are specially invited from those who are engaged in any kind of philanthropical work. A new edition of the leaflet describing the purposes of the Bureau of Information is now ready and will be sent free of charge on application.**

### Approved Home.

We have pleasure in adding the following Home to our Approved List :—

Liskeard, Cornwall.—5 Westwood. Principal : Miss Constance Burton, Certificated Nurse and Masseuse. Medical, surgical, maternity, chronic, and convalescent patients received. Terms from £2 2s.

### Home Required for Phthisical Case.

A home is wanted, preferably in a southern county, for a girl of eleven years, suffering from consolidation of the upper half of the left lung with cavity at apex, where open-air treatment could be provided, and, if necessary, tuberculin treatment given. A weekly payment of 15s. to 18s. would be made by a lady interested in the case. If any of our Approved Homes can receive this case they are requested to communicate with C.H., 165a.

---

### *THE INSTITUTIONAL HOUSEKEEPER.*

### Food Transportation.

A very efficient food-carrier is in use at one of the American hospitals. It consists of a metal case or box, supported on large rubber-tyred wheels. The case is constructed in such a way as to form a non-conducting jacket, the space between the lining and the outer cover being packed with non-conducting vegetable fibre. The food vessels are of aluminium, each of which fits into its special compartment provided in the jacketed case. By its use food can be transported from the main kitchen to the remotest part of the building without apparent loss of heat; it is lighter than the water-jacketed type, and required less attention. In the hospital from which the design emanated, a carrier is provided for each serving room, and the food is served from it direct to the trays, thus reducing handling to a minimum.

### *THE INSTITUTIONAL ARTIFICER.*

### QUESTIONS FOR JUNE.

**1. Describe and, if possible, give a sketch or photograph of any home-made piece of hospital furniture or apparatus you have designed, or which you have found to serve a useful purpose at your institution.**

**2. Describe any defects you have found in your heating and hot-water system, and the steps you have taken to remedy them.**

*NOTE.—A minimum payment of FIVE SHILLINGS will be made for each descriptive note based upon the foregoing questions which is accepted for publication.*

RULES.

The following rules must be observed:—

1. Contributions must be written on one side of the paper. They must bear the name and address of the sender and be accompanied by coupon to be cut from the back cover (inside page) of the current issue of The Hospital. A pseudonym must be chosen if the name is not to be published.

2. They must be addressed to the Editor of The Hospital, 28 & 29 Southampton Street, Strand, London, W.C., so as to reach him before the end of the month, and must be marked in the left-hand corner "Institutional Artificer."

### *EMPLOYMENT AND TRAINING.*

### Schools for Mothers.

The main object of these institutions is to reduce the high rate of infantile mortality—so generally prevalent among the poorer classes, and especially in evidence in the industrial areas of population, where many young mothers are drawn from the factories and the mills, and possess little or no experience of domestic life—by instructing the mothers in their duties to themselves as mothers, and generally in the proper care of their infants. This branch of social work is of comparatively recent development, and during the past two or three years many such schools or institutions having similar objects have been established in various towns and districts throughout the country. Some of these are administered by societies specially formed for the purpose, others by existing health societies and medical charities, and a few by municipal authorities. The local medical officers of health and the health visitors are generally prominently associated with the work. The form of instruction given necessarily varies in accordance with the facilities provided in the schools.

Infant Consultations.

The essential features are the "Infant Consultations," which the mothers are expected to attend at least once a fortnight for instruction and for the inspection of their infants; and systematic home-visiting, by which means the mothers may be helped to apply what is taught in the class-room. Some schools hold classes on infant care and feeding, cooking, cutting out or making clothing, and general hygiene; and a few provide cheap dinners for nursing or expectant mothers. The work is largely performed by volunteers, but salaried officers are employed in a certain number of schools. The establishment of these schools and the closer relationship of those interested in the work is promoted by the Association of Infant Consultations and Schools for Mothers, which was formed under the auspices of the National League for Physical Education and Improvement, 4 Tavistock Square, London, W.C. This League has issued a very interesting and instructive pamphlet, based upon statistics gathered from a large number of institutions undertaking this work.—"M. W."

### Nursing Institutions.

There are a number of public and private institutions in London which employ nurses on the co-operative system; one of the most important is the Nurses' Co-operation, 8 New Cavendish Street, London, W. You will do well to study the advertisements which appear in *The Nursing Mirror* from time to time. In Birmingham you might apply to the Nurses' Co-operation, 278 Monument Road, Edgbaston, and to the Trained Nurses' Co-operation and Hostel, 54 Park Hill, Moseley, near Birmingham. When applying for particulars enclose a stamped addressed envelope for reply. Full particulars of similar institutions are incorporated in the book "How to Succeed as a Nurse," which is now in course of publication, and may be obtained of The Scientific Press, Ltd., 28-29 Southampton Street, Strand, London, W.C.

### *MISCELLANEOUS.*

### Books on Nursing.

We do not know of any books which deal especially with the nursing aspects of hip and spine disease. You will find a study of either of the following helpful:—"Common Disorders of Childhood," by Dr. S. F. Still, price 16s. net, and "Diseases of Children," by Goodhart and Still, 15s. Both these are largely used by nurses, but they do not treat mainly of the diseases in question. In Cheyne and Burghard's "Manual of Surgical Treatment," vol. iii., you will find a very full account of both diseases; this is an expensive work, however, the price of the volume being one guinea. All these may be obtained of The Scientific Press, Ltd., 28-29 Southampton Street, Strand, London, W.C.—"M. M. C."

### EDITOR'S LETTER-BOX.

A Gramophone for the Aran Isles.

Inishmain, Isles of Aran,
Galway Bay.
June 16.

Dear Editor.—I write to see whether I could put in a little appeal for a gramophone in your paper, The Hospital? It is the only one that reaches me here in this lonely and isolated place, far away from civilisation.

This island is the loneliest and most inaccessible of the Aran group. We have no amusements, and the joys and comforts of the mainland are unobtainable here. In winter it is exceptionally wild and the loneliness unbearable. Perhaps some kind reader would send us a second-hand gramophone. I am sure a great many people outside are quite tired of them now, and this would be quite an innovation to our islanders.—I am, dear Editor, sincerely yours,

(Nurse) B. P. Hedderman,
District Nurse to the Aran Isles.

*Case of Advanced Phthisis.—M. E. F.* We are making inquiries and hope to hear of some home or institution able to assist this case. We gather from your remarks that the patient is insured; can you say whether the local Insurance Committee have indicated their inability to provide sanatorium treatment?

"*Sirod.*"—We are glad to learn that the information we gave you was helpful. Should you decide to purchase the home we shall be pleased to hear from you in the matter of registration.

*A. C.*—We are glad to hear that you are in communication with a home, and trust that you will make a satisfactory arrangement; we are forwarding further communications.

*Miss Eastty.*—We thank you for your communication; we have noted your new address in our Register.

*Communications have been received from the following and duly forwarded*:—Miss L. Starey, Miss R. G. Campbell, Miss M. Aldridge, Miss Pearmain, Miss Haslock, Miss Greene, Mrs. Day, Mrs. Ambler, Miss Hilda Wood, and Mr. M. E. A. Martin.

The Editor will be glad to receive correspondence and to consider contributions upon all subjects relating to institutional work, and affecting the welfare of institutional officers.

### EDITOR'S NOTICES.

**Contributions**

Contributions should be written, or preferably typed, on one side of the paper only, and all articles sent in are accepted upon the distinct understanding that they are forwarded to The Hospital only.

The Editor cannot undertake to return MSS. not used, but when a stamped directed envelope is enclosed the MSS. may be returned if a special request is made.

Accepted articles and paragraphs of news will be paid for after publication at the scale rate.

**Address**

To prevent delay all contributions and letters on editorial business must be addressed exclusively to the Editor, "The Hospital," 29 Southampton Street, Strand, London, W.C. It is important that this regulation shall be strictly observed.

**Correspondence:**

Correspondence on all subjects is invited. The name and address of correspondents must be given as a guarantee of good faith, but not necessarily for publication.

**Special Articles:**

Special articles are invited, and questions, inquiries, and paragraphs upon all matters relating to the work, administration and management of general, special, mental, fever, cottage and convalescent hospitals, sanatoria, homes, institutions, societies and organisations for the treatment or care of the sick, injured and dependants of all classes. Every matter affecting the interests and welfare of the staff of all grades working in these institutions will receive special consideration.

### HANDBOOKS
### For TRAINED WORKERS

POCKET·GUIDE SP SERIES

**1/- net. 1/1 Post free.**

**Bound in Limp Cloth.**
**Suitable Size for the Pocket.**

The works which comprise the S.P. Pocket Guide Series are intended for ready reference, consequently the aim of the Publishers has been to make them as concise and lucid as possible. Each book is written by an expert on the subject of which it treats, and the utmost reliance therefore can be placed in the information it imparts.

**Essentials of Fever Nursing.** By LYTTON MAITLAND, M.D. (Lond.), M.B., B.S., D.P.H. (Camb.).

**Manual and Atlas of Swedish Exercises.** (*With over 60 Illustrations.*) By THOMAS D. LUKE, M.D., F.R.C.S.

**Bandaging Made Easy.** (*Illustrated with over 90 Diagrams.*) By M. HOSKING, Sister-in-Charge, Tredegar House, Bow, E.

**How to Write and Read Prescriptions.** By LYTTON MAITLAND, M.D. (Lond.), M.B., B.S., D.P.H. (Camb.).

**Principal Drugs and their Uses.** By A PHARMACIST.

**Asepsis and How to Secure It.** By H. W. CARSON, F.R.C.S.

**Treatment after Operations.** *Rules for Nursing after General and Special Operations.* By MARY WILES.

**The Nurse's Duties before and during Operations.** By MARGARET FOX, Matron, Prince of Wales's General Hospital, Tottenham.

**Other works, in amplification of this Series, will be announced in due course.**

*Published by*

**The SCIENTIFIC PRESS, Ltd.**

**28/29 Southampton Street, STRAND, LONDON, W.C.**

## Figures and Tables

**Figure f1:**
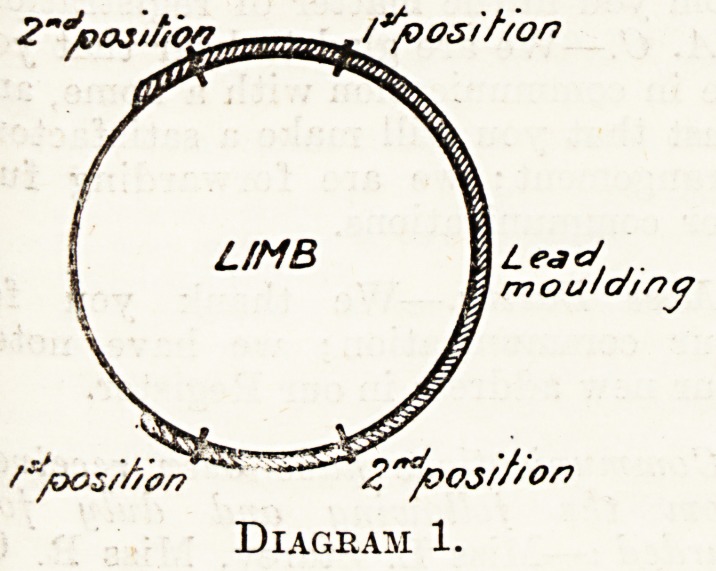


**Figure f2:**